# Supplementary material for: Play Activities Are Associated with Force Regulation in Primary School
Source: J Funct Morphol Kinesiol. 2024 Mar 18;9(1):54. doi: 10.3390/jfmk9010054 (PMC10971395; doi:10.3390/jfmk9010054)
Supplement: Supplementary file 1 [file jfmk-09-00054-s001.zip › jfmk-2841970-supplementary.pdf]

Table S1. Effects of total quantity of exercise experience on CFE

	TQEE Low [*n*=8]	TQEE Middle [*n*=8]	TQEE Large [*n*=7]	ANCOVA		Effect size f
	Mean ± SD	Mean ± SD	Mean ± SD	F -value	*p*-value	0.561
CFE (%)	1329.9 ± 547.6	1075.0 ± 253.6	1067.9 ± 302.4	2.83	0.085	

covariance = Age, Maximum grip strength

ANCOVA, analysis of covariance; CFE, Controlled Force Exertion; TQEE, total quantity of exercise experience
